# 1208. Impact of Selected Antimicrobial Stewardship and Infection Control Strategies in Carbapenem Resistance in a Third Level Public Hospital in Colombía.

**DOI:** 10.1093/ofid/ofad500.1048

**Published:** 2023-11-27

**Authors:** G E R A R D O A MUÑETON, C A R L O S A SOLORZANO, J U L E I M A CARDENAS, E L E N A V CASTRO, J E S S I C A L MORALES

**Affiliations:** Hospital MIlitar Central - Hospital de Kennedy, BOOGOTA D.C, Distrito Capital de Bogota, Colombia; UNIVERSIDAD NACIONAL DE COLOMBIA, BOOGOTA D.C, Distrito Capital de Bogota, Colombia; UNIVERSIDAD COOPERATIVA DE COLOMBIA, BOOGOTA D.C, Distrito Capital de Bogota, Colombia; UNIVERSIDAD DE LOS ANDES, BOOGOTA D.C, Distrito Capital de Bogota, Colombia; UNIVERSIDAD NACIONAL DE COLOMBIA, BOOGOTA D.C, Distrito Capital de Bogota, Colombia

## Abstract

**Background:**

Antimicrobial Stewardship (AMS) Programs and Infection prevention and control (IPC) strategies have been shown to effectively reduce antimicrobial resistance. The resources required for the implementation of these programs in low-resource settings could be limited. This study aimed to assess the impact of a few AMS and IPC strategies on carbapenem resistance in a third-level public hospital in Colombia.

**Methods:**

A retrospective before-and-After study was completed. The baseline period consisted of 6 months (January 2021-Jun 2021) and the intervention period consisted of 18 months (July 2021-December 2022) The AMS intervention consisted of the implementation of prospective audits on prescriptions of meropenem, cefepime, and piperacillin-tazobactam, along with educational seminars. The IPC intervention consisted of screening for carbapenemase-producing-Enterobacterales (CPE) through rectal swabs for every patient transferred to the ICU and weekly for all patients in the ICU. A cohorting area with 24 beds was also established to isolate CPE colonized/infected patients

**Results:**

During the intervention period, 886 prospective audits were made. We suspended 208, 111, and 55 prescriptions of Meropenem, piperacillin-tazobactam, and cefepime respectively. The length of therapy was shortened in 239, 290, and 99 prescriptions of Meropenem, piperacillin-tazobactam, and cefepime respectively.

The antimicrobial consumption was lower in the intervention period for meropenem (24,8 DDD/100 patients-days Vs 74.3 DDD/100 patients-days baseline period). Figure 1

The prevalence of positive rectal swabs showed a downward trend during the intervention period. Figure 2. The prevalence of CPE also decreased and was 0,45 during the baseline period and 0,28 during the last semester of the intervention period. Figure 3

The carbapenem resistance *K. pneumoniae* laboratory-confirmed bloodstream infection rate also decreased during the intervention period, going from 15.4/10.000 patients-days to 1.17/10.000 patient-days. Figure 4

**Figure:**
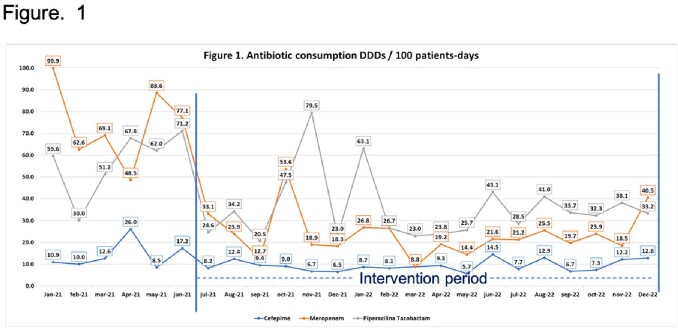

**Figure:**
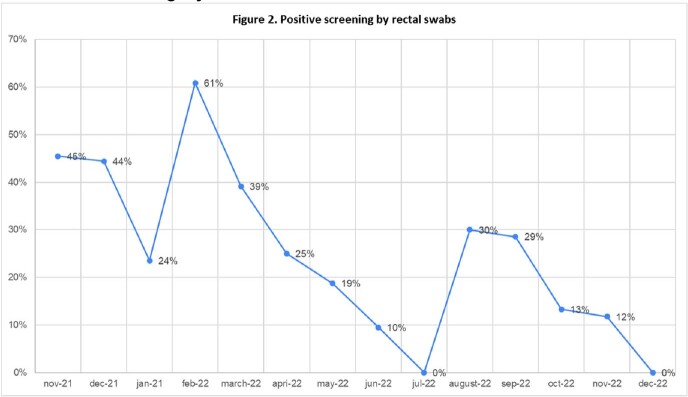

**Figure:**
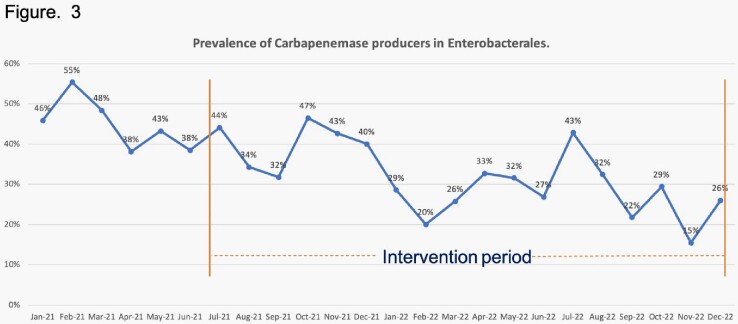

**Conclusion:**

The implementation of selected AMS and IPC strategies in a resource limited third level hospital reduced the prevalence of carbapenemase-producing Enterobacterales and the rate of Carbapenem-resistant *K. pneumoniae* bloodstream infections.

**Figure:**
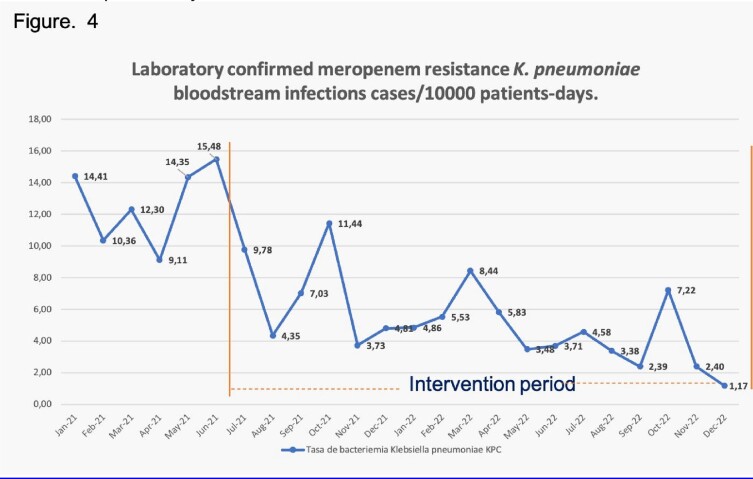

**Disclosures:**

**GERARDO A. MUÑETON, Infectious diseases and Internal Medicine Specialist**, PFIZER: Advisor/Consultant

